# Federated Learning in Endodontics: A Framework for Privacy-Preserving Multicentre Artificial Intelligence

**DOI:** 10.1016/j.identj.2026.109704

**Published:** 2026-06-22

**Authors:** Mohammed Turky, Lakshman Samaranayake, Thanaphum Osathanon, Paul M.H. Dummer

**Affiliations:** a Department of Endodontics, Faculty of Dentistry, Minia University, Minia, Egypt; b Department of Endodontics, Faculty of Dentistry, Sphinx University, Assiut, Egypt; c Center of Excellence in Periodontal Disease and Dental Implantology, Faculty of Dentistry, Chulalongkorn University, Bangkok, Thailand; d Faculty of Dentistry, University of Hong Kong, Hong Kong, Hong Kong; e Global Research Cell, Dr D.Y. Patil Dental College and Hospital, Dr D.Y. Patil Vidyapeeth, Pimpri, Pune, India; f Center of Artificial Intelligence and Innovation (CAII) and Centre of Excellence for Dental Stem Cell Biology, Faculty of Dentistry, Chulalongkorn University, Bangkok, Thailand; g School of Dentistry, College of Biomedical and Life Sciences, Cardiff University, Cardiff, UK

**Keywords:** Artificial intelligence, Data privacy, Endodontics, Federated learning

## Abstract

**Introduction and Aim:**

High-quality artificial intelligence (AI) models in endodontics require access to diverse, well-annotated datasets. This review introduces federated learning (FL) as a privacy-preserving framework for collaborative AI in endodontics.

**Methods:**

In this narrative review, a comprehensive literature search was conducted across databases, encompassing studies published up to April 2026. The search strategy was intentionally broad to facilitate a thorough exploration of the evolution of the relevant concepts. However, to ensure consistency in comprehension and analysis, the review was limited to publications in English. Studies describing the fundamentals and applications of FL were reviewed and comparatively analysed. The narrative review served as the framework for outlining implementation pathways, challenges, and research priorities for its application to diagnostic and decision-support tasks.

**Results:**

Traditional centralised training methods face legal and ethical challenges due to data protection regulations. FL allows institutions to retain local patient data while contributing model updates to a central server or decentralised network, thus providing a viable alternative. The article explores FL principles, privacy and security mechanisms, architectures, technical challenges, and adversarial risks. Regulatory and ethical considerations hinge on a mix of advanced technical measures and robust organisational practices. A proposed roadmap for implementing FL includes pilot studies, standardised data processes, clinical validation, and regulatory engagement. FL promises to enhance AI development in endodontics while safeguarding patient privacy, with potential benefits in diagnostics and personalised care.

**Conclusion:**

FL could advance AI integration in endodontics, prioritising the protection of patient privacy. This initiative holds the potential to improve diagnostic processes and facilitate personalised treatment approaches.

**Clinical Relevance:**

FL enables the development of multicentre AI models without sharing patient data. By leveraging diverse clinical datasets, this approach may improve the accuracy and generalisability of AI systems for endodontic diagnosis, treatment planning, and outcome prediction while preserving patient privacy.

## Introduction

Artificial intelligence (AI) has swiftly become a pivotal element in transforming the healthcare landscape, fundamentally altering diagnostic methodologies, enabling personalised treatment paradigms, and enhancing clinical decision-making through data-driven analytics.^1^ This evolution is also evident in dentistry, where the incorporation of AI technologies is progressively advancing the field. Significant advances have been made in areas such as radiographic interpretation, caries detection, orthodontic assessments, and endodontic diagnoses, highlighting the potential of AI to improve clinical outcomes and enhance patient care.^2^, ^3^, ^4^ However, maximising the potential of AI in healthcare is contingent upon access to large, diverse, and high-quality datasets.^5^ This challenge is further complicated by stringent data-protection regulations, institutional silos that impede collaboration, and ethical concerns surrounding the centralisation of sensitive health information.^6^ These obstacles could be particularly pronounced in endodontics, where the fragmentation of datasets across clinics, hospitals, and geographic locations significantly undermines the generalisability and robustness of AI models.^7^

Federated learning (FL) has recently emerged as a compelling solution to these challenges encountered by the healthcare sector.^8^ Initially conceptualised by Google in 2016, FL enables multiple healthcare institutions to collaboratively train machine-learning models without sharing raw patient data, thereby preserving patient privacy.^9^ Instead of exchanging sensitive information, institutions share only model parameters or gradients, facilitating collective learning while strictly adhering to privacy laws such as the General Data Protection Regulation in Europe and the Health Insurance Portability and Accountability Act in the United States.^10^

In endodontics, the need for privacy-preserving collaboration is particularly significant. Complex diagnostic tasks such as identifying periapical pathology, assessing fracture lines in teeth, predicting treatment outcomes, and determining the suitability of cases for vital pulp therapy or retreatment benefit greatly from access to large and heterogeneous datasets that accurately reflect real-world clinical variability.^11^ However, compiling such datasets centrally is exceedingly challenging due to the regulatory and logistical barriers.

The feasibility of FL has already been validated in several medical domains, particularly in radiology and oncology, where it has enabled improved diagnostic performance and patient management.^12^^,^^13^ Despite these advances, the adoption of FL in dentistry, and specifically in endodontics, remains limited. To date, only a small number of dental studies have explored federated approaches, and none have comprehensively addressed their application to the unique diagnostic, prognostic, and treatment-planning challenges encountered in endodontic practice.^14^ This gap highlights the need for a systematic evaluation of the potential role, advantages, limitations, and future directions of FL in endodontic care.

The purpose of this article is to provide a comprehensive description of FL to enable key stakeholders to understand its potential application in endodontics. Specifically, this review evaluates the relevance of FL to the field, outlines its potential benefits for clinicians and healthcare systems, examines both the technical and ethical challenges associated with its implementation, and proposes future research pathways to translate into clinical practice.

## Methods

Due to the nascent and evolving nature of the field of FL within endodontics, a narrative review was chosen for this work to enable a more comprehensive integration of various perspectives – technological, clinical, ethical, and infrastructural – and to offer a critical interpretation of the current early-stage literature. This type of review is particularly advantageous for hypothesis-building and framework development, which are essential for positioning FL as a transformative paradigm for future research and clinical innovation in endodontics. By adopting this approach, we can effectively synthesise fragmented evidence, contextualise its relevance to the field of endodontics, and articulate a coherent agenda for advancing future empirical studies. This narrative framework will not only catalyse further inquiry but also foster interdisciplinary collaboration that is essential for integrating FL into routine endodontic practice.

The authors collaboratively drafted an outline that encompassed a comprehensive list of major topics to be explored within the paper. This outline underwent thorough scrutiny and revisions from all contributing authors to ensure clarity and alignment of focus. Following this initial phase, an electronic search was performed to gather pertinent literature from a variety of reputable databases, including PubMed/MEDLINE, Scopus, Web of Science, IEEE Xplore, and Google Scholar for studies released up to April 2026.

The search strategy deployed a strategic combination of specific keywords, including ‘artificial intelligence’, ‘data privacy’, ‘dentistry’, ‘distributed machine learning’, ‘endodontics’, ‘federated analytics’, ‘federated learning’, ‘root canal’, and ‘secure aggregation’. Notably, the search was unrestricted by a specific date range, allowing for a more comprehensive understanding of the evolution of these concepts; however, it was narrowed to publications strictly in English to maintain uniformity in comprehension and analysis.

The initial search results were meticulously screened based on the relevance of titles and abstracts, ensuring that only the most pertinent and focused research was considered. Subsequently, full-text articles that appeared to hold significant relevance were subjected to a detailed review, and those ultimately deemed valuable were included in the narrative synthesis. Moreover, their reference lists were checked in an attempt to include relevant studies manually to ensure a comprehensive understanding of the topic.

While formal inclusion and exclusion criteria were not strictly applied, a clear focus was maintained on several key aspects: the role of FL in endodontic practices, its advantages over traditional approaches, the challenges it faces in practical implementation, privacy and governance issues, and prospective future directions for its integration into endodontic care.

The main topics explored in this review are: What is FL?, Benefits of implementing FL in endodontics, technical and statistical challenges in FL, privacy, governance, and regulatory considerations, roadmap for translational implementation in endodontics, integration with explainable AI (XAI) and clinical workflows, research agenda, and priorities.

## Discussion

### What is FL?

FL operates as a distributed machine-learning framework in which multiple clients, such as hospitals, dental clinics, and imaging centres, collaboratively train a shared global model under the coordination of a central server. This computational framework enables collaborative model development by aggregating locally trained model updates from distributed nodes and subsequently redistributing the updated global model^15^ (Figure).

**Fig:**
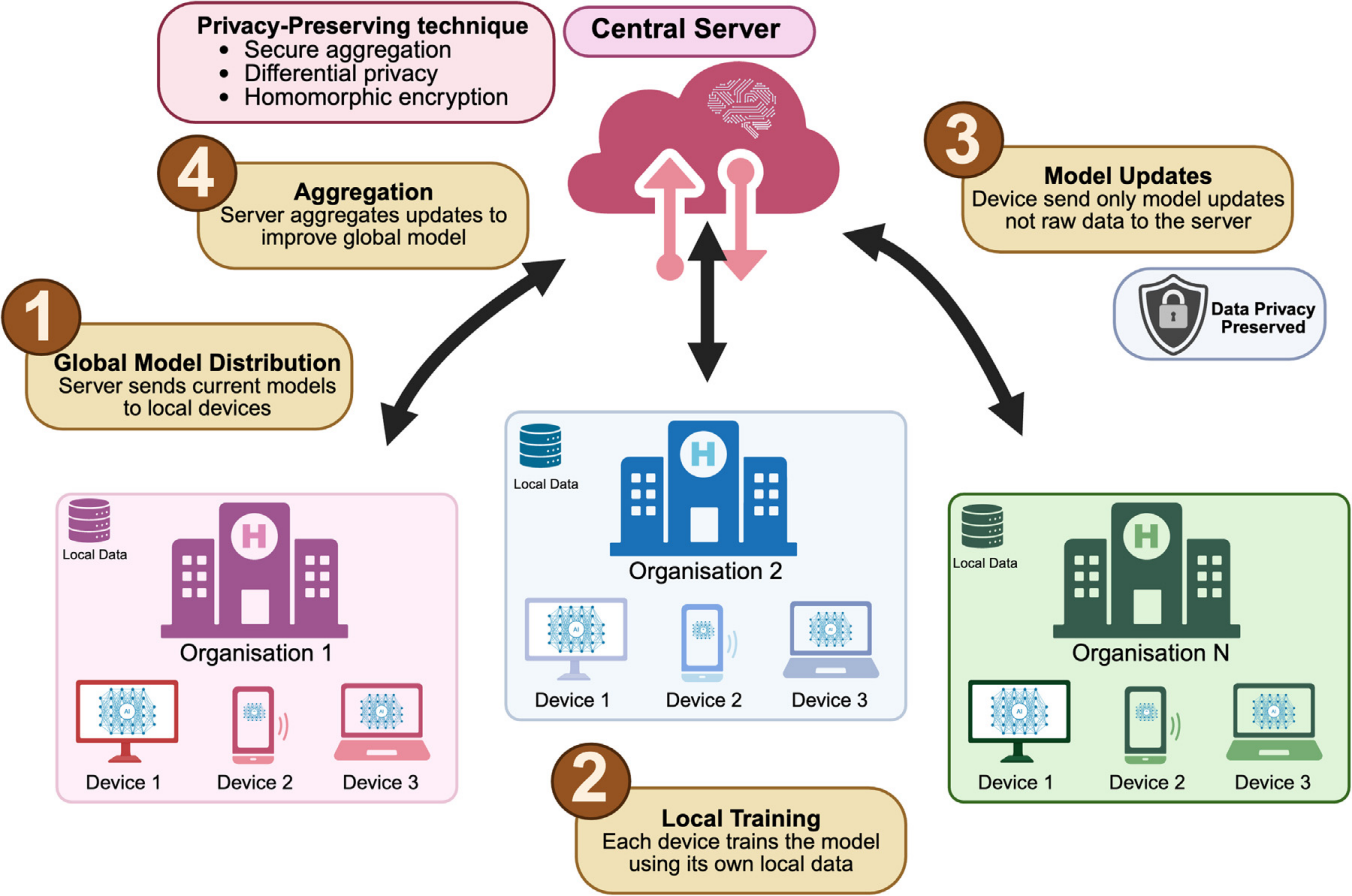
*Federated learning (FL) concept.* FL operates as a distributed machine-learning framework that works together to train a shared global model. The central server sends the model to local devices (1), and the devices train the model using their own local data (2). The distributed nodes then send model updates, *not raw data,* back to the central server (3), which aggregates the updates to improve the global model (4). This virtuous cycle is repeated until the model fully converges. Created in BioRender. Osathanon, T. (2026) http://BioRender.com/3srlh82.

Federated averaging (FedAvg) is the foundational algorithm in FL, in which individual clients compute local model updates using their respective datasets. These updates, which contain gradients or weights, are then transmitted to a server that aggregates the information, typically via weighted averaging. The resulting updated global model is redistributed for subsequent training rounds.^16^

FL can be categorised into several configurations to accommodate different data distribution characteristics: (1) *Horizontal FL* involves clients working with the same feature space but across different patient populations; (2) *vertical FL* applies when clients share overlapping patient cohorts but possess different feature sets; (3) *federated transfer learning* is used when both the feature spaces and patient populations differ across institutions.^17^

To enhance data security, various techniques can be utilised within FL frameworks.^18^ For example, secure aggregation methods protect the server from accessing individual client updates. Differential privacy introduces carefully calibrated noise to updates, minimising the risk of reidentification. Additionally, homomorphic encryption enables operations on encrypted data, though this comes at the cost of increased computational requirements.

In endodontic applications, FL enables learning patterns across heterogeneity of imaging datasets, thereby enhancing the external validity of the developed models. In addition, the diverse demographics and clinical practices in multicentre cohorts foster the development of more accurate and reliable predictive models for endodontic treatment outcomes. By training AI models in a federated manner across diverse populations, FL enables the identification and reduction of potential biases inherent in machine learning datasets. Furthermore, integrating XAI methodologies into FL pipelines can yield interpretable, locally relevant insights, thereby enhancing clinicians’ decision-making processes. Through these applications, FL stands at the forefront of AI innovation for endodontics, promising to enhance diagnostic accuracy, treatment planning, and overall patient outcomes while addressing critical privacy and security challenges.

### Benefits of implementing FL in endodontics

FL may offer significant advantages for clinicians, institutions, and health systems by enabling the creation of AI models trained on expansive, diverse datasets without the need to transfer sensitive imaging data. For dentists, specifically endodontists, FL enhances the models’ ability to generalise across various real-world conditions, including differences in imaging equipment, disease presentations, and demographic factors such as ethnicity.^19^ As a result, endodontists gain more robust and reliable clinical decision support that bolsters diagnostic accuracy.^20^ FL enables endodontists to access the latest AI advancements more quickly.^14^ This rapid integration of innovations circumvents the prolonged wait associated with developing national datasets, which can take years or even decades.

For institutions and stakeholders, FL minimises legal, ethical, and cybersecurity risks related to centralised data storage, enabling smaller centres to actively participate in extensive AI development.^10^ FL could democratise innovation by enabling smaller clinics or academic institutions without extensive datasets to contribute to and benefit from advanced AI research projects. This inclusivity fosters collaboration and knowledge-sharing across the healthcare landscape, including dentistry and endodontics in particular, accelerating research initiatives at both national and global levels.^19^

At the health-system level, FL reduces infrastructure costs, facilitates collaborative research across geographic boundaries, and helps avert costly treatment failures by bolstering early diagnosis and informed decision-making. Thus, FL has the potential to deliver tangible clinical, operational, and economic value throughout the spectrum of endodontic care.

### Technical and statistical challenges in FL

#### Client-count and sample-size considerations in FL

The performance and effectiveness of FL are intricately linked to two critical factors: the number of participating clients and the volume and quality of data each client contributes.^21^ The general principle is that increasing the number of participating centres can enhance the model’s generalisability. This is achieved by exposing the learning algorithm to a broader spectrum of variables, including diverse imaging devices, varying operator techniques, and a wide range of patient demographics.^21^ However, it is important to note that the benefits derived from increasing the number of centres may eventually plateau if individual centres provide only very small datasets. In such instances, the contribution of each centre may not significantly influence the overall performance of the model.

On the other hand, a smaller cohort of well-curated centres that each possess sufficiently large and high-quality local datasets could potentially outperform a larger network comprised of centres with sparse or inconsistent data contributions.^22^ For a pilot study focusing on endodontics, a pragmatic approach would entail involving approximately 3 to 5 well-selected centres, each contributing several hundred labelled cases, such as conventional periapical radiographs, cone-beam computed tomography (CBCT) scans, or structured treatment records. This level of data contribution is generally adequate to evaluate the feasibility of the FL approach, establish effective communication workflows, and observe preliminary convergence behaviour in model training.

However, it is crucial to recognise that more complex deep-learning tasks – such as three-dimensional segmentation of CBCT images or predictive modelling of treatment outcomes – would likely necessitate the involvement of larger multicentre cohorts, as well as multiple rounds of training to optimise model performance.

Furthermore, the convergence of the model is influenced by several factors beyond just sample size. Key elements include class balance (the uniform distribution of different classes within the dataset), annotation consistency (the reliability and accuracy of the labels assigned to data), feature heterogeneity (the diversity of features used across different data sources), and the degree of similarity or dissimilarity between the participating sites.

Given these complexities, early-stage FL studies in the field of endodontics should prioritise the harmonisation of data – ensuring that the data formats, labels, and features are consistent across different sites. Additionally, a focus on establishing realistic pilot-scale collaborations is recommended before expanding to larger consortia. Once the technical feasibility of the FL framework has been demonstrated through these initial studies, it would then be appropriate to consider scaling up to include a broader network of participating centres.

#### Statistical heterogeneity

In the realm of FL, data across participating sites are often nonindependent and identically distributed, posing a significant statistical challenge.^23^ Differences in patient demographics, imaging modalities, device calibration, and clinical workflows lead to heterogeneity in label prevalence, image quality, and feature distributions. Such variability can impair model convergence and compromise global performance.

Mitigation strategies include personalised FL techniques that enable client-specific fine-tuning, robust aggregation methods that adapt to local variations, and federated domain adaptation techniques designed to harmonise intersite variability while preserving decentralisation.^23^ Addressing statistical heterogeneity is essential to ensure reliable generalisation in real-world clinical settings.

#### Label inconsistency and annotation standards

Label inconsistency represents an additional limitation. Interrater variability in radiographic interpretation can be significant, even within a single institution,^24^ potentially propagating bias across federated models. This inconsistency underscores the need for harmonised annotation protocols. Employing consensus-based strategies, such as multireader labels or adjudication processes, can enhance consistency and ensure higher fidelity in data labelling. Establishing harmonised diagnostic criteria across institutions and standardised protocols is fundamental to ensuring robust supervised learning within federated frameworks.

#### Communication and computational constraints

The transmission of large images (cephalometric and panoramic radiographs, facial photographs, intraoral scanning data, and CBCT scans) necessitates efficient communication strategies in federated settings.^25^ To mitigate the burden of high communication costs, techniques such as adaptive update scheduling, through quantisation and sparsification, are essential. Efficient workload distribution across sites is equally important to prevent bottlenecks arising from heterogeneous computational resources. Without careful optimisation, communication costs may limit scalability in multicentre networks.

#### Security and adversarial risks

Despite decentralisation, FL systems remain vulnerable to adversarial manipulation. Malicious clients can compromise model integrity via model poisoning or ‘backdoor attacks’ while gradient inversion techniques can potentially reconstruct sensitive information from shared updates.^26^ To combat these threats, employing robust aggregation techniques (eg, trimmed-mean or median-based approaches), anomaly-detection methods, secure aggregation protocols, and differential privacy measures can strengthen the system against potential vulnerabilities.^27^

#### Evaluation and validation

Robust evaluation frameworks are critical for clinical translation and generalisability. Cross-site validation using hold-out datasets at each participating centre, combined with independent external test cohorts, provides a rigorous assessment of generalisability.^28^ Such evaluation strategies are essential to assess the model’s reliability and applicability across diverse populations.

### Privacy, governance, and regulatory considerations

While FL can help reduce privacy risks, it does not eliminate them.^29^ Model updates may still leak sensitive information if they are not adequately protected. Accordingly, a comprehensive privacy-preserving FL deployment must integrate technical, organisational, and legal safeguards^29^^,^^30^: (1) *Technical safeguards:* Implementing secure aggregation, differential privacy, and encrypted communication protocols. (2) *Organisational safeguards:* Establishing formal data-user agreements, governance oversight, maintaining audit trails, and strict data minimisation. (3) *Legal safeguards:* Demand adherence to jurisdiction-specific frameworks and regulations, and establish clear patient consent protocols, along with ongoing engagement with institutional ethics and review bodies. Increasingly, regulators require rigorous validation, demonstrable clinical performance, and postmarket surveillance for AI-based medical systems.

### Roadmap for translational implementation in endodontics

Successful implementation of FL in endodontics requires a staged pragmatic approach strategy that prioritises feasibility, governance, and clinical validation.

#### Phase 0 – Stakeholder alignment

Establish a multi-institution, transnational consortium of academic dental hospitals and schools, large referral practices, imaging vendors, data scientists, and patient representatives. The primary focus should be on defining common objectives, establishing governance structures, and developing data standards, including specifications for imaging formats, DICOM/CBCT settings, and metadata schemas to ensure interoperability across sites.

#### Phase 1 – Feasibility pilot

Initiate a proof-of-concept federated project targeting a clearly defined task, such as automated detection of periapical radiolucency across centres. Key deliverables should include the development of a secure federated training pipeline, implementation of secure aggregation protocols, standardised annotation and quality-control procedures, and a predefined evaluation framework using local hold-out datasets. This phase should assess technical feasibility, model convergence under heterogeneous conditions, and operational constraints.

#### Phase 2 – Multisite model development

Scale the network to encompass additional imaging centres while incorporating robust aggregation strategies and differential privacy mechanisms to address statistical heterogeneity and privacy risk. This phase should actively integrate clinician feedback and provide explanations for model predictions through XAI techniques.

#### Phase 3 – Prospective clinical validation

Deploy the model prospectively within participating clinics to evaluate diagnostic performance, impact on clinical decision-making, workflow integration, and user acceptance. Performance metrics should extend beyond accuracy to include calibration, subgroup analyses, and real-world robustness across imaging systems and patient demographics.

#### Phase 4 – Regulatory submission and adoption

Prepare comprehensive technical and clinical documentation to support regulatory review. Parallel development of postimplementation surveillance mechanisms is essential to monitor performance drift, update governance frameworks, and ensure ongoing compliance with evolving regulatory standards.

To enhance the clinical relevance of FL in endodontics, several specific applications should be thoroughly explored. One immediate and practical use case is the detection of periapical lesions using both conventional periapical radiographs and CBCT scans. By implementing multicentre federated models, we can improve the robustness of the diagnostic process across various imaging devices and exposure protocols that may differ from one clinic to another. This cross-institutional collaboration has the potential to increase diagnostic accuracy and reliability by integrating diverse data sources without compromising patient privacy.

Similarly, FL can significantly aid in the automated identification of vertical root fractures – conditions that are often challenging to detect and require a variety of radiographic datasets to ensure reliable model training. This approach could streamline diagnosis and enhance the quality of patient care by providing clinicians with advanced tools that draw on a wider knowledge base.

In the realm of treatment planning, FL systems may play a crucial role in estimating working lengths by analysing radiographic images in conjunction with data obtained from integrated apex locators. This collaborative method could lead to improved precision in treatment preparations while ensuring that sensitive institutional data is kept secure and private.

Another promising application of FL within endodontics lies in anatomical classification tasks. For instance, recognising complex canal systems, such as C-shaped canals or other intricate morphologies, can be quite challenging. By pooling learning data from geographically diverse populations, federated models can better capture anatomical variations and intricacies than what is typically available from single-centre datasets.

Beyond diagnosis and treatment planning, FL can enable the development of prognostic models aimed at predicting outcomes for various endodontic treatments, including root canal treatment, retreatment, or vital pulp therapy. By leveraging distributed longitudinal clinical records, along with radiographic follow-up and procedural variables, clinicians can gain deeper insights into treatment efficacy and patient outcomes.

Collectively, these examples underscore the notion that FL in endodontics represents more than just a theoretical solution for privacy concerns. It provides a practical framework for fostering collaborative intelligence across various facets of the clinical workflow, including diagnosis, anatomical recognition, treatment execution, and outcome forecasting. Embracing FL can ultimately advance the field of endodontics, leading to improved patient outcomes and more personalised treatment strategies.

Table summarises the potential applications of FL in endodontics, illustrating key clinical tasks, relevant data modalities, and suitable FL configurations (horizontal, vertical, or transfer learning) for privacy-preserving multicentre collaboration.

**Table:** The potential applications of FL in endodontics, relevant data modalities, and suitable FL configurations (horizontal, vertical, or transfer learning) for privacy-preserving multicentre collaboration.

Endodontic task	Primary data modality	Example FL configuration	Rationale
Periapical lesion detection	Periapical radiographs/CBCT	Horizontal FL	Multiple centres hold similar imaging data with different patients
Vertical root fracture detection	Periapical radiographs/CBCT	Horizontal FL	Requires rare-case aggregation across institutions
Working length determination	Periapical radiographs + apex locator signals + clinical metadata	Vertical FL	Different data types may exist across collaborating sites
C-shaped canal recognition	CBCT/preoperative imaging	Horizontal FL	Multipopulation anatomical variation improves generalisation
Root canal treatment outcome prediction	Clinical records + imaging + follow-up data	Transfer FL/hybrid FL	Centres differ in variables, size, and case-mix
Retreatment prognosis	Prior treatment records + imaging	Transfer FL	Smaller datasets can benefit from pretrained federated models
Vital pulp therapy success prediction	Pulp status, symptoms, radiographs, follow-up	Vertical or transfer FL	Multimodal predictors distributed across systems

CBCT, cone-beam computed tomography; FL, federated learning.

### Integration with XAI and clinical workflows

To enhance clinician trust and adoption in endodontics, FL models should be integrated with XAI tools that provide clear local explanations.^31^^,^^32^ For instance, in radiographic assessments, heatmaps can visually highlight areas of concern, while feature importances can elucidate the decision-making process within prognostic models. This integration must prioritise low-latency inference by enabling local deployment of the global model, ensuring that AI system responses are prompt and relevant in clinical scenarios. Additionally, seamless integration with Picture Archiving and Communication Systems is essential for the smooth operation of these AI tools in clinical workflows. Finally, human-in-the-loop frameworks should be established to allow clinicians to review, contextualise, and override AI-generated suggestions. Such oversight preserves professional autonomy, reinforces accountability, and supports responsible integration of federated AI systems into endodontic practice.

### Research agenda and priorities

To advance FL models in endodontic imaging, a targeted research agenda is imperative. Developing standardised benchmark datasets specifically for endodontic imaging would catalyse innovation and enhance the reproducibility of results among researchers. To enhance methodological rigour and reproducibility in future FL research within endodontics, prioritising the establishment of benchmark datasets and external validation frameworks, alongside aligning study design and reporting practices with recognised AI research guidelines – such as CLAIM for imaging studies, TRIPOD + AI for predictive modelling, and CONSORT-AI for interventional or clinical evaluations – will significantly bolster transparency, allow for better comparability, and facilitate the translation of findings into clinical dental practice.

Organised federated challenges can stimulate interest and encourage collaborative solutions in tackling diverse clinical problems. In addition, it is essential to develop robust methods for managing label noise and heterogeneity, such as federated uncertainty estimation and consensus labelling techniques. These approaches can help improve the reliability of datasets gathered from different sources, ultimately enhancing model performance.

Further, investigating AI models’ robustness to adversarial attacks and designing certified defences tailored to the unique characteristics of dental imaging modalities is crucial. This work can help ensure that AI applications remain secure and trustworthy in operational settings. Understanding the economic impact is vital for encouraging widespread adoption among practitioners. Conducting thorough economic analysis and implementation studies allows stakeholders to quantify the costs, benefits, and barriers associated with integrating AI into routine endodontic practice. Lastly, ethical studies should be conducted to assess patient attitudes towards the use of federated models, especially regarding their expectations for transparency in AI decision-making processes. Engaging patients in this dialogue can foster trust and acceptance.

## Conclusion

FL offers a viable and strategically important framework for multicentre AI development, significantly reducing the need to share raw patient data. This not only addresses critical privacy concerns but also navigates the regulatory complexities inherent in healthcare systems. FL could be particularly valuable for analysing endodontic images, such as periapical radiographs and CBCT scans, and for constructing prognostic models that benefit from geographically and demographically diverse datasets. This diversity is essential for improving the generalisability of AI models. The efficacy of privacy guarantees hinges on integrating advanced technical solutions, such as secure aggregation and differential privacy, with organisational safeguards and strong governance frameworks.

Such a holistic approach is vital to maintaining trust in AI applications. Key technical challenges must be systematically addressed, including the management of statistical heterogeneity, ensuring label consistency, and mitigating adversarial risks. Robust engineering practices and thoughtful study design are critical in overcoming these hurdles.

A pragmatic translational roadmap, comprising conducting pilot federated experiments, standardising data processing pipelines, clinical validation efforts, and active regulatory engagement, is essential for the widespread adoption of FL. With rigorous design and interdisciplinary collaboration, FL could ultimately support the development of clinically robust, privacy-preserving AI systems capable of advancing evidence-based endodontic care.

## Declaration of generative AI and AI-assisted technologies in the writing process

During the preparation of this manuscript, the authors used a generative artificial intelligence tool to assist with language refinement, structural organisation, and clarity of expression. The authors critically reviewed and edited all AI-assisted content and take full responsibility for the accuracy, integrity, and originality of the final manuscript.

## Data availability

Data sharing is not applicable to this article as no datasets were generated or analysed during the current study.

## Author contributions

Mohammed Turky: Conceptualisation, methodology, formal analysis, investigation, resources, writing – original draft preparation. Lakshman Samaranayake: Formal analysis, writing – review, and editing. Thanaphum Osathanon and Paul M.H. Dummer: Formal analysis, writing – review and editing.

## Conflict of interest

LS was supported by the Chulalongkorn University, Second Century (C2) High-Potential Professoriate Fund at its Faculty of Dentistry. Other authors declare that they have no known competing financial interests or personal relationships that could have appeared to influence the work reported in this article. The author is an Editorial Board Member/Editor-in-Chief/Associate Editor/Guest Editor for this journal and was not involved in the editorial review or the decision to publish this article.
